# Stress Responses Elicited by Glucose Withdrawal in *Aspergillus fumigatus*

**DOI:** 10.3390/jof8111226

**Published:** 2022-11-21

**Authors:** Tamás Emri, Károly Antal, Barnabás Gila, Andrea P. Jónás, István Pócsi

**Affiliations:** 1 Department of Molecular Biotechnology and Microbiology, Faculty of Sciences and Technology, University of Debrecen, Egyetem tér 1, 4032 Debrecen, Hungary; 2 ELRN-UD Fungal Stress Biology Research Group, Egyetem tér 1, 4032 Debrecen, Hungary; 3 Department of Zoology, Eszterházy Károly Catholic University, Eszterházy tér 1, 3300 Eger, Hungary; 4 Doctoral School of Nutrition and Food Sciences, University of Debrecen, Egyetem tér 1, 4032 Debrecen, Hungary

**Keywords:** *Aspergillus fumigatus*, carbon stress, chitin degradation, glutathione metabolism, iron limitation, oxidative stress, RNA sequencing

## Abstract

Glucose is a widely used carbon source in laboratory practice to culture *Aspergillus fumigatus*, however, glucose availability is often low in its “natural habitats”, including the human body. We used a physiological–transcriptomical approach to reveal differences between *A. fumigatus* Af293 cultures incubated on glucose, glucose and peptone, peptone (carbon limitation), or without any carbon source (carbon starvation). Autolytic cell wall degradation was upregulated by both carbon starvation and limitation. The importance of autolytic cell wall degradation in the adaptation to carbon stress was also highlighted by approximately 12.4% of the *A. fumigatus* genomes harboring duplication of genes involved in N-acetyl glucosamine utilization. Glucose withdrawal increased redox imbalance, altered both the transcription of antioxidative enzyme genes and oxidative stress tolerance, and downregulated iron acquisition, but upregulated heme protein genes. Transcriptional activity of the Gliotoxin cluster was low in all experiments, while the Fumagillin cluster showed substantial activity both on glucose and under carbon starvation, and the Hexadehydro-astechrome cluster only on glucose. We concluded that glucose withdrawal substantially modified the physiology of *A. fumigatus*, including processes that contribute to virulence. This may explain the challenge of predicting the in vivo behavior of *A. fumigatus* based on data from glucose rich cultures.

## 1. Introduction

*Aspergillus fumigatus*, as a saprophytic mold, occurs widely in our environment. Their conidia are inhaled frequently and in substantial quantities [1]. Inhaled conidia pose a risk for immunocompromised and immunodeficient people, and, at high concentrations, even for healthy individuals [2]. In fact, *A. fumigatus* is the leading cause of several diseases, including noninvasive aspergillomas, allergic bronchopulmonary aspergillosis, chronic pulmonary aspergillosis, chronic necrotizing pulmonary aspergillosis, severe asthma with fungal sensitization, invasive aspergillosis, and extrapulmonary aspergillosis [3]. In the case of invasive aspergillosis, the case fatality rate is high, usually between 30 and 95% [4], and the increasing prevalence of drug-resistant strains have a further negative impact [5].

The survival and success of fungi within the human body depend largely on how effectively they can adapt to this special habitat. They have to tolerate the temperature and pH of our tissues; they have to cope with oxidative, nitrosative, or transition metal stresses initiated by our immune system, and with the possible presence of antifungal drugs used during prophylaxis or treatment; and they also have to adapt to oxygen, iron, zinc, and carbohydrate (glucose) limitations [6,7]. Previous studies have demonstrated that stress tolerance against a specific stressor is highly dependent on culturing conditions (e.g., temperature, pH, available nutrients) and on other stressors acting either simultaneously or sequentially with the studied stressor (combinatorial stress) [7,8]. For example, growing in a glucose rich medium, under iron limitation, or under nitrosative or ionic stress all increased the oxidative stress tolerance of the studied fungus [7,8]. Therefore, studies based on standard (glucose rich) laboratory conditions and single stressor treatments miss important details on how *A. fumigatus* survives in our body. A stressor that is deleterious in vitro may have negligible effect in vivo due to different environmental conditions as well as its combination with various other stresses.

Due to its filamentous nature, *A. fumigatus* has to cope with glucose withdrawal stress in two situations: (1) When the hyphal tips (or germinating conidia) grow into an area with low glucose availability, hyphae have to maintain their growth under this stress. (2) When the colony has exhausted glucose around the subapical regions of the hyphae, hyphae have to continue differentiation and conidia production in the absence of glucose. The two different aims (i.e., maintaining vegetative growth and reproductive differentiation) are not necessarily achieved by the same strategy. Upon low glucose availability in its environs, the colony—in order to find better habitats—can increase radial growth with decreased biomass production [9]. This process is usually supported by the utilization of weak carbon sources (e.g., plant cell wall materials) if they are available in the medium, and most likely by macroautophagy reutilization of materials from subapical regions. In addition to consumption of weak carbon sources, macroautophagy (resulting in “empty hyphae”) and autolytic cell wall degradation (utilization of cell wall biopolymers of “empty hyphae”) can provide nutrients for developmental processes [9,10].

Although glucose is widely used at a 10–20 g/L concentration as a carbon source in the laboratory, its availability is much lower in soil, compost, and many other “typical” habitats of *A. fumigatus*, including the human body where blood glucose levels are usually less than 0.1 g/L. In addition to this “glucose limitation stress”, *A. fumigatus* also has to cope with carbon starvation stress in the human body (e.g., in the phagolysosome). Growing on a carbon energy source other than glucose or surviving in the absence of any (external) carbon energy source substantially alters the morphology, ultrastructure and physiology of fungi and necessarily affects their stress tolerance and virulence [7,11]. Here, we studied carbon starvation (induced by the absence of any carbon source) and carbon limitation (induced by glucose withdrawal in the presence of casein peptone) stress responses of *A. fumigatus*. In addition to understanding how the fungus adapted to these conditions, we focused on how glucose withdrawal influences assumed virulence traits such as iron metabolism, antioxidative processes, and secondary metabolism of this human pathogen.

## 2. Materials and Methods

### 2.1. Strain, Culturing Conditions

The *Aspergillus fumigatus* Af293 strain was studied. It was maintained on Barratt’s minimal agar plates [12]. In all experiments, conidia freshly isolated from 6 d cultures incubated at 37 °C were used for inoculation.

During submerged cultivation, 100 mL aliquots of Barratt’s minimal broth supplemented with 5 g/L yeast extract (in 500 mL Erlenmeyer flasks) were inoculated with 5 × 10^7^ conidia/flask and were incubated at 37 °C and with a 220 rpm shaking frequency for 16 h to reach the exponential growth phase. In the case of physiological experiments, mycelia from each flask were transferred into 100 mL fresh Barratt’s minimal broth containing either 4 g/L casein peptone (Sigma–Aldrich Ltd., Budapest, Hungary) as a carbon energy source, or did not contain any carbon source. All these cultures were incubated at 37 °C and 220 rpm and samples were taken at 0 d, 1 d, 2 d, and 5 d.

In the case of transcriptomics studies, mycelia were transferred into 100 mL fresh Barratt’s minimal broth containing no carbon source (“carbon-starved”), or either 10 g L^−1^ glucose (“glucose”), or 10 g/L glucose and 4 g/L casein peptone (“glucose + peptone”), or 4 g/L casein peptone (“peptone”) as a carbon energy source. These cultures were also incubated at 37 °C and 220 rpm. To study stress-adapted cultures, samples were taken for RNA isolation at 24 h (carbon-starved and peptone cultures). Due to the fast utilization of glucose, samples were taken at 4 h in the case of cultures containing glucose.

### 2.2. Agar Plate Assays

Sensitivity of the strain to oxidative and iron chelation stresses was tested on Barratt’s minimal agar plates containing 10 g/L glucose, or 10 g/L glucose and 4 g/L casein peptone, or 4 g/L casein peptone (“peptone”) as carbon energy source and also supplemented with either menadione sodium bisulfite (MSB; 0 or 0.04 mM) as an oxidative stress inducing agent or deferiprone as iron chelator (DFP; 0 or 3 mM). Plates were point inoculated with 5 μL conidia suspension containing 10^5^ conidia and cultures were incubated at 37 °C for 5 day. Stress sensitivity was characterized with growth reduction relative to untreated cultures. In the iron chelation stress experiments, the media did not contain trace element solution.

### 2.3. Detecting Growth and Metabolic Activity

For submerged cultures, we characterized growth by changes of dry cell mass (DCM), and metabolic activity by methylthiazoletetrazolium (MTT) reduction, as described in Emri et al. [13].

### 2.4. Enzyme Assays

Extracellular N-acetyl-glucosaminidase [14], β-glucosidase [15], and protease [16] activities were determined from the fermentation broth, while γ-glutamyl transpeptidase (γGT) [17] activities were measured both from the fermentation broth and the cell-free extract prepared by X-pressing [18].

### 2.5. Measuring Redox Imbalance, GSH, and GSSG Contents

The carbon stress-induced redox imbalance was determined with 2′,7′-dichlorofluorescein (DCF-assay), as described earlier [19]. Changes in the reduced and oxidized glutathione (GSH and GSSG, respectively) content of hyphae were measured with the DTNB-glutathione reductase assay [20] from cell-free extracts prepared by 5-sulfosalicylic acid treatment [21].

### 2.6. Reverse-Transcription Quantitative Real-Time Polymerase Chain Reaction (RT-qPCR) Assays

RT-qPCR assays were carried out with a Luna^®^ universal one-step RT-qPCR kit (New England Biolabs, Ipswich, MA, USA), following the manufacturer’s protocols, using the primer pairs listed in Table S1 and total RNA was isolated from lyophilized mycelia according to Chomczynski (1993) [22]. Relative transcription levels were characterized with the difference between the crossing points of the reference and target gene within a sample (ΔCP). Since the traditional “housekeeping” genes showed high-transcriptional changes under carbon stress, we selected—based on preliminary assays and the RNAseq experiments—Afu3g14240 (putative tRNA splicing protein) as our reference gene.

### 2.7. High-Throughput RNA Sequencing

RNA sequencing (from library preparation to generation of fastq.gz files) was carried out at the Genomic Medicine and Bioinformatic Core Facility, Department of Biochemistry and Molecular Biology, Faculty of Medicine, University of Debrecen, Debrecen, Hungary. Total RNA was isolated from lyophilized mycelia using three biological replicates. Single-read 75 bp Illumina sequencing was performed [23]. Library pools were sequenced in the same lane of a sequencing flow cell, and 17.2–26.7 million reads per sample were obtained. Quality control of the reads was performed with the FastQC package (Babraham Bioinformatics; http://www.bioinformatics.babraham.ac.uk/projects/fastqc; accessed on 15 November 2022). More than 96% of reads (in the case of each sample) were successfully aligned to the genome of *A. fumigatus* Af293 with the hisat2 software (version 2.1.0) (Daehwan Kim Lab.; http://daehwankimlab.github.io/hisat2/download/; accessed on 15 November 2022). Differentially expressed genes were determined with DESeq2 (version 1.24.0) (Bioconductor; https://bioconductor.org/packages/release/bioc/html/DESeq2.html; accessed on 15 November 2022).

### 2.8. Detecting Allele-Specific Expression

The expression of the Afu8g04070, Afu8g04080, Afu8g04090, Afu8g04100, Afu8g04110, Afu8g04120, and Afu8g04130 genes and their paralogues (Afu1g00480, Afu1g00470, Afu1g00460, Afu1g00450, Afu1g00440, Afu1g00420, and Afu1g00410, respectively) was studied as follows: For each gene pair and RNA sequencing sample we counted the number of reads exactly matching only the first gene, only the second gene, or both (biopython-1.79, https://biopython.org/; accessed on 15 November 2022). Exact matches with only one of the genes, were taken as an indicator of that gene was probably being expressed. Transcript sequences were downloaded from FungiDB (https://fungidb.org/fungidb/app; accessed on 15 November 2022).

### 2.9. Detecting Gene Duplications in A. fumigatus Genomes

Duplication of Afu8g04070-Afu8g04130 genes were tested in 194 *A. fumigatus* genomes as follows: Paired end samples were downloaded using Ena File Downloader (version:1.0.6, ENA; https://ena-docs.readthedocs.io/en/latest/retrieval/file-download.html#using-ena-file-downloader-command-line-tool; accessed on 15 November 2022) for the accessions investigated (Table S1), aligned to the genome of *A. fumigatus* Af293 (NCBI; https://ftp.ncbi.nlm.nih.gov/genomes/refseq/fungi/Aspergillus_fumigatus/latest_assembly_versions/GCF_000002655.1_ASM265v1/GCF_000002655.1_ASM265v1_genomic.fna.gz; accessed on 15 November 2022) using Bowtie2 version 2.3.5.1 (Source Forge; https://sourceforge.net/projects/bowtie-bio/files/bowtie2/2.3.5.1/; accessed on 15 November 2022) [24], and sorted by coordinates (Picard Command Line SortSam, version 2.18.25, Broad Institute; https://broadinstitute.github.io/picard/; accessed on 15 November 2022). The resulting alignments were processed with FREEC-11.6 (Boeva Lab.; http://boevalab.inf.ethz.ch/FREEC/; accessed on 15 November 2022) using 0.08 for the breakpoint threshold and 10,000 bases for the window size [25]. Based on the overlaps between the copy number variation (“gain” or “loss”) regions obtained from FREEC and the regions of investigated genes we counted the number of accessions with “gain” or “loss” affecting each gene.

### 2.10. Evaluation of Transcriptome Data

When two transcriptomes were compared (“A” vs. “B”), upregulated and downregulated genes were defined as genes showing significantly different expressions (adjusted *p*-value < 0.05; Deseq2) and |log_2_FC| > 1, where FC (fold change) stands for the number calculated by the DESeq2 software using “B” as the reference transcriptome.

Composition of upregulated or downregulated gene sets was characterized with gene set enrichment analyses using the ShiniGo platform (South Dakota State University; bioinformatics.sdstate.edu/go/; accessed on 15 November 2022) and applying default settings. Only hits with a corrected *p*-value < 0.05 were regarded as significantly enriched.

The “fisher.test” function of R project (Fisher’s exact test; R Project; www.R-project.org/; accessed on 15 November 2022) was used to study the enrichment of the following gene groups in the upregulated and downregulated gene sets:

“CAZyme” (carbohydrate-active enzyme) genes are genes that were collected from the carbohydrate-active enzymes database (CAZy; http://www.cazy.org/; accessed on 15 November 2022).

“Heme binding protein” genes are genes belonging to the “Heme binding” FunCat term (FungiFun2; https://elbe.hki-jena.de/fungifun/; accessed on 15 November 2022; [26]).

The gene groups “Heme biosynthesis” genes, “Fe-S cluster protein” genes, and “Fe-S cluster assembly” genes are described in Kurucz et al. [27].

“Fe acquisition” genes are putative and known siderophore metabolism genes, reductive iron assimilation (RIA) genes, other Fe^2+^ transporter or pump genes, and transcription factor genes according to Haas [28] and Kurucz et al. [27].

“Autophagy related” genes are putative and known “autophagy” and “autophagic” protein genes according to the ShiniGo platform (South Dakota State University; bioinformatics.sdstate.edu/go/; accessed on 15 November 2022).

“Chitinase” genes are putative and known chitinase genes according to the ShiniGo platform (South Dakota State University; bioinformatics.sdstate.edu/go/; accessed on 15 November 2022).

“Chitine utilization” genes are orthologues of *Aspergillus nidulans* putative N-acetyl glucosamine transporter, N-acetylglucosamine-6-phosphate deacetylase, and glucosamine-6-phosphate deaminase genes (AN1427, AN1428, and AN1418, respectively).

“Glucanase” genes are putative and known glucanase genes collected from the ShiniGo platform (South Dakota State University; bioinformatics.sdstate.edu/go/; accessed on 15 November 2022), but omitting the β-1,4- and β-1,3-1,4-glucanase genes.

“Secreted peptidase” genes are putative and known peptidase genes collected from the ShiniGo platform (South Dakota State University; bioinformatics.sdstate.edu/go/; accessed on 15 November 2022) but keeping only those where the description suggests the secretion of the protein.

“Glutathione degradation and synthesis” genes are putative GSH synthase, γ-glutamyl-cysteine ligase and γGT genes according to the ShiniGo platform (South Dakota State University; bioinformatics.sdstate.edu/go/; accessed on 15 November 2022), as well as orthologues of *Saccharomyces cerevisiae* and *A. nidulans* DUG (GSH degrading) pathway genes (*DUG1*, *DUG2*, *DUG3*, and *dugA*, *dugB*, *dugC*, respectively).

“Antioxidative enzyme” genes are known and putative superoxide dismutase (SOD), catalase, peroxidase, glutathione/glutaredoxin/thioredoxin redox system genes collected from the ShiniGo platform (South Dakota State University; bioinformatics.sdstate.edu/go/; accessed on 15 November 2022).

“Secondary metabolite cluster” genes are manually or experimentally determined secondary metabolite cluster genes collected by Inglis et al. [29] and Lin et al. [30]. In this case, gene set enrichment analysis was carried out with each secondary metabolite cluster separately.

## 3. Results

### 3.1. Carbon Stress-Induced Hydrolase Secretion and Increased Redox Imbalance in A. fumigatus

**Figure 1:**
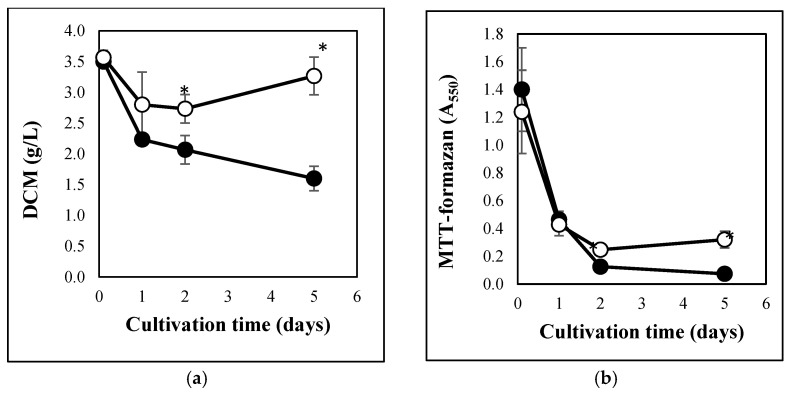
Changes in the DCM (**a**) and MTT reducing activity (**b**) of carbon-limited and carbon-starved *A. fumigatus* cultures. *A. fumigatus* Af293 was grown in complex mediums, and mycelia from exponentially growing phase cultures were resuspended in carbon-source-free minimal mediums either supplemented with 4 g/L casein peptone (carbon-limited cultures; ○) or not (carbon-starved cultures; ●). Mean ± SD calculated from three parallel experiments are presented. *—Significant differences between the mean values of the carbon-starved and carbon-limited cultures at the marked time point according to the Student’s *t*-test (*p* < 0.05).

**Figure 2:**
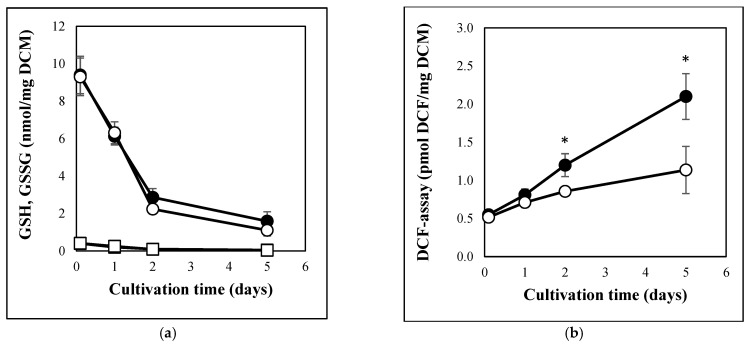
Changes in the GSH and GSSG content (**a**) as well as in the DCF producing activity (**b**) of carbon-limited and carbon-starved *A. fumigatus* cultures. Exponentially growing phase *A. fumigatus* Af293 cultures were resuspended in carbon-source-free minimal mediums either supplemented (carbon-limited cultures; ○ for GSH and DCF-assay, □ for GSSG) or not (carbon-starved cultures; ● for GSH and DCF-assay, ■ for GSSG) with 4 g/L casein peptone. Mean ± SD calculated from three parallel experiments are presented. *—Significant differences between the mean values of the carbon-starved and carbon-limited cultures at the marked time point according to the Student’s *t*-test (*p* < 0.05). Note, the GSSG levels of the two cultures were substantially not different.

**Figure 3:**
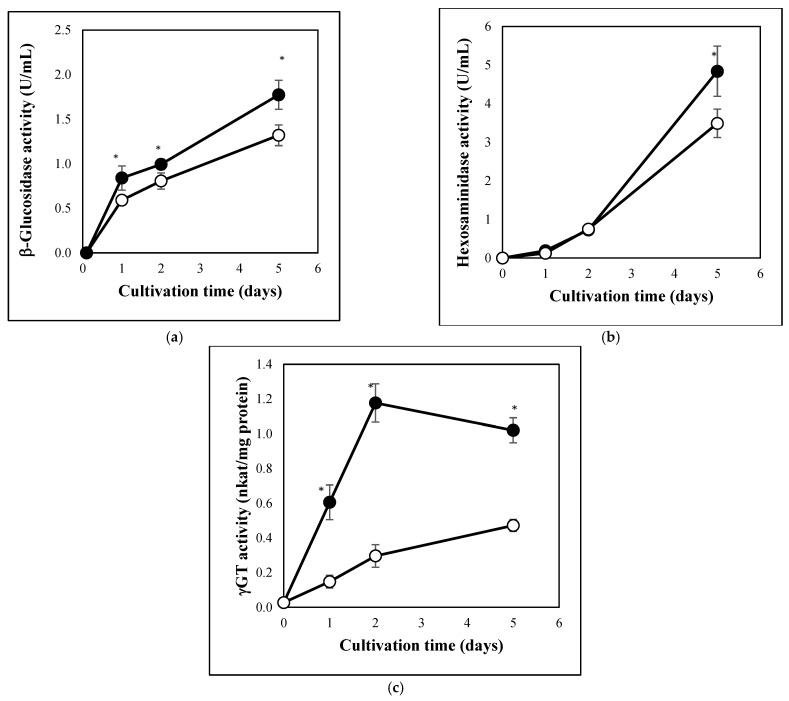
Changes in the extracellular β-glucosidase (**a**), extracellular hexosaminidase (**b**) and intracellular γGT (**c**) activities of carbon limited and carbon-starved *A. fumigatus* cultures. *A. fumigatus* Af293 mycelia from exponentially growing phase cultures were resuspended in carbon-source-free minimal mediums either supplemented (carbon-limited cultures; ○) or not (carbon-starved cultures; ●) with 4 g/L casein peptone. Mean ± SD calculated from three parallel experiments are presented. One unit (U) was defined as an amount of enzyme that can cause a one-unit change in the absorbance over 1 h under the conditions described by the method used. *—Significant differences between the mean values of the carbon-starved and carbon-limited cultures at the marked time point according to the Student’s *t*-test (*p* < 0.05).

Carbon stress caused similar physiological changes in *A. fumigatus* cultures to those observed previously in *A. nidulans* or *P. chrysogenum* [18,19]. Carbon starvation decreased both DCM and MTT reducing activity of the cultures (Figure 1). These changes were accompanied with the partial depletion of GSH pools (Figure 2a), increasing redox imbalance (Figure 2b), secretion of extracellular hydrolases (Figure 3a,b), and an increase in intracellular γGT activity (Figure 3c). From the cell-free fermentation broth, we could detect considerable β-glucosidase and hexosaminidase activities (Figure 3a,b), but not a substantial amount of secreted protease (detected with azocasein), nor secreted γGT (detected with γ-glutamyl-*p*-nitroanilide) activities. In contrast to the carbon-starved cultures, MTT reducing activity and DCM decreased between 0 d and 2 d but thereafter seemed to increase (Figure 1). Smaller redox imbalances (Figure 2b) as well as reduced β-glucosidase, hexosaminidase, and γGT productions (Figure 3) were observed in peptone supplemented cultures than under carbon starvation. The changes in the GSH and GSSG pools were similar to those observed in carbon-starved cultures (Figure 2a) and no extracellular protease, or extracellular γ-glutamyl tranferase activities were detected.

### 3.2. Glucose Withdrawal Had Substantial Consequences on the Transcriptome; Peptone Modified the Transcriptome Markedly Only in the Absence of Glucose

**Figure 4:**
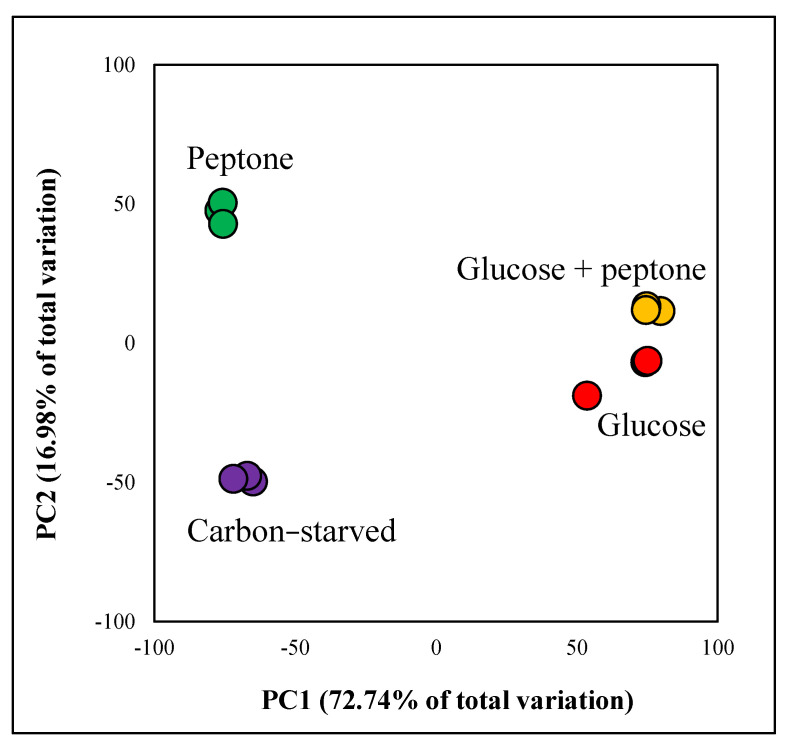
Principal component (PC) analysis of the transcriptome data. The analysis was carried out using the rlog data generated by DESeq2 software.

**Figure 5:**
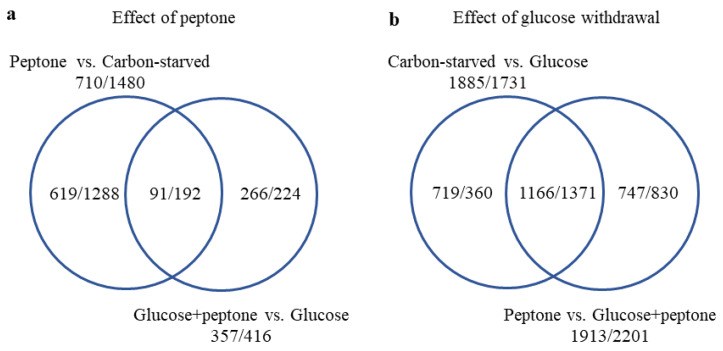
Distribution of genes upregulated and downregulated in the presence of peptone (**a**) or in the absence of glucose (**b**).

To investigate the physiological consequences of glucose withdrawal, we compared the transcriptomes of the following four cultures: cultures grown on glucose (“glucose”), on glucose and peptone (“glucose + peptone”), on peptone (“peptone”), and in the absence of any carbon source (“carbon-starved”). Transcriptional changes recorded by RNA sequencing showed a good positive correlation with data obtained by RT-qPCR (Table S2). According to the principal component analysis of the transcriptome data (Figure 4) as well as to the Venn diagram of the upregulated and downregulated genes (Figure 5), glucose had a substantial effect on the transcriptome and the effect of peptone was highly dependent on the presence of glucose; it was weak on glucose, but stronger in the glucose-free cultures.

Gene set enrichment analyses suggested that glucose withdrawal downregulated processes involved in the degradation of glucose (e.g., glycolysis, TCA cycle, respiration) and bulk protein synthesis (e.g., translation, amino-acid, and ribonucleotide synthesis) in line with the observed inhibition of the growth (Figure 1), while it upregulated amino-acid and polysaccharide catabolism (Table 1 and Table S3). The downregulation of several other processes such as ergosterol, fatty acid (lipid), cell wall (glucan), and vitamin biosynthesis, as well as sulfate, nitrate, phosphate, and iron acquisition (Table 1 and Table S3), may be also related to the observed growth inhibition (Figure 1).

In the absence of glucose, peptone downregulated polysaccharide catabolism (however the genes involved in the utilization of N-acetylglucosamine were upregulated), but amino-acid catabolism was not downregulated (Table 2 and Table S3). The upregulation of ribosome biogenesis genes (Table 2 and Table S3) was in line with the increased growth observed relative to the carbon-starved cultures (Figure 1).

In the presence of glucose, peptone also upregulated ribosome biogenesis as well as ergosterol biosynthesis (Table 2 and Table S3). Substantial changes in nitrogen metabolism were also observed: nitrate utilization was downregulated (together with reactive nitrogen species metabolic processes) and alterations in amino-acid metabolism were detected (Table 2 and Table S3).

The transcriptional behavior of potentially interesting gene sets was also evaluated (Table 3, Table 4 and Table 5 and Table S4) and summarized in the forthcoming paragraphs.

### 3.3. Autolytic Cell Wall Degradation Is Important in Carbon Stress Adaptation

Genes related to (macro)autophagy were not enriched in any upregulated or downregulated gene sets (Table 3 and Table S4). However, vacuolization and intensive formation of “empty hyphae” as typical consequences of macroautophagy were observed under carbon stress (Figure S1). In contrast, vacuolization and “empty hypha” formation was accompanied with the enrichment of upregulated autophagy-related genes in carbon-starved *A. nidulans* cultures [31,32]. This discrepancy may be due to our use of stress-adapted cultures here, while Gila et al. [31,32] recorded an early stress response. A quick increase in the abundance of a protein during the early stress response may need a higher mRNA pool than maintaining the abundance at a high level later [33].

We observed the upregulation of genes potentially involved in autolytic cell wall degradation after glucose withdrawal (Table 3 and Table S4). When comparing carbon-starved and “glucose” cultures, glucanase genes (encoding known or putative α- and β-1,3-glucanases) showed enrichment in both the upregulated and downregulated gene sets (Table 3 and Table S4). This dual behavior concurs with the results of Gila et al. [31], who studied transcriptional changes in *A. nidulans*, and supports the view that some glucanases are involved in cell wall degradation and others in cell wall biogenesis. Nevertheless, Afu1g04260 (*ENGL1*), the orthologue of *A. nidulans engA* β-1,3-endoglucanase gene involved in autolytic cell wall degradation in that species [34], was upregulated by carbon starvation (Tables S2 and S4). Although chitinase genes were not enriched after glucose withdrawal in the upregulated gene sets, five chitinase genes, including Afu8g01410 (*chib1*, an orthologue of *A. nidulans chiB* chitinase gene involved in autolytic cell wall degradation; [35]) showed upregulation during carbon starvation (Table 3 and Tables S2 and S4). Importantly, N-acetylglucosamine-6-phosphate deacetylase, glucosamine-6-phosphate deaminase, and N-acetyl glucosamine transporter genes (“chitin utilization genes”) were all upregulated in the absence of glucose (Table 3 and Tables S2 and S4). These transcriptional changes concur well with the observed decreases of DCM (an indicator of autolytic cell wall degradation; [36]) (Figure 1a) and an increase in secreted hexosaminidase activities (Figure 3b). In the presence of peptone both chitinase and glucanase genes were enriched in the downregulated gene set, while surprisingly, chitin utilization genes were enriched in the upregulated gene set (Table 3 and Table S4).

The N-acetyl glucosamine transporter, N-acetylglucosamine-6-phosphate deacetylase, and glucosamine-6-phosphate deaminase genes exist in duplicated forms in the genome of *A. fumigatus* Af293 [37]. The Afu8g04070 (putative glucosamine-6-phosphate deaminase), Afu8g04100 (putative N-acetylglucosamine-6-phosphate deacetylase), and Afu8g04110 (putative N-acetyl glucosamine transporter) genes are part of a gene set containing seven genes far from the telomeres of chromosome 8. This gene set also occurs on chromosome 1, close to the telomere. Both gene sets were transcriptionally active (Table S5). In the *A. fumigatus* A1163 strain, this gene set occurred only on scaffold seven (equivalent to chromosome 8 of Af293) between two genes that were orthologues of the genes surrounding the gene set on chromosome 8 in Af293 (Figure S2). Testing 194 *A. fumigatus* genome accessions against the Af293 genome, FREEC indicated no change in the copy number of the seven studied genes, including the three N-acetylglucosamine utilization genes of chromosome 8 (Table S6). Twenty-four accessions also contained the chromosome 1 part of the gene set either partly (18 accessions) or completely (six accessions) (Table S6). Thus, the studied region was at least partly duplicated in 12.4% (24/194) of the accessions.

### 3.4. GSH Depletion Was Accompanied with Upregulation of γGT, but Not DUG Pathway Genes

During carbon starvation of fungi, macroautophagy and autolytic cell wall degradation are accompanied by degradation of the intracellular GSH pool as well as an intensive search for extracellular proteins and plant cell wall polymers (i.e., upregulation of extracellular peptidase and CAZyme genes) [11,31,32,36].

The transcription of CAZyme genes was substantially affected by the availability of glucose and peptone (Table 3 and Table S4). In both peptone vs. carbon-starved and peptone + glucose vs. glucose culture comparisons there were many more downregulated CAZyme genes than upregulated CAZyme genes (Table 3 and Table S4). In contrast, glucose withdrawal upregulated several genes, while also downregulating several others similar to earlier results in *A. nidulans* [32]. When comparing peptone vs. glucose + peptone, both upregulated and downregulated genes were enriched in the appropriate gene set (Table 3 and Table S4).

Although carbon starvation upregulated genes were involved in the utilization (degradation) of amino acids (Table 2) as well as certain peptidase genes (e.g., *sedC*, *pep2*, *cp7*, *alp1*, *mep20*, *sedE*; Table S4), the absence of glucose and the presence of peptone in glucose-free cultures resulted in the enrichment of secreted peptidase genes in the downregulated gene set (Table 3 and Table S4). In line with this result, we could not detect any protease activity in the cell-free fermentation broth.

The Afu7g04760 gene, the orthologue of *A. nidulans ggtA* responsible for dual extra- and intracellular γGT production, did not show upregulation in *A. fumigatus*, however another putative γGT gene, Afu4g13580, was able to show upregulation (Tables S2 and S4). This upregulation is in line with the recorded increase in intracellular γGT activities of carbon-stressed cultures (Figure 3c). Although the DUG pathway genes (*DUG1*, *DUG2*, *DUG3* in *Saccharomyces cerevisiae* and *dugA*, *dugB*, *dugC* in *A. nidulans*) are involved in the degradation of cytosolic GSH under stress [31,38] and the GSH pools decreased in glucose-free cultures (Figure 2b), the DUG gene orthologues did not show upregulation in our experiments (Table S4).

### 3.5. Antioxidative Enzymes and Secondary Metabolism Cluster Genes Had Transcriptional Patterns Characteristic of the Carbon Source

**Figure 6:**
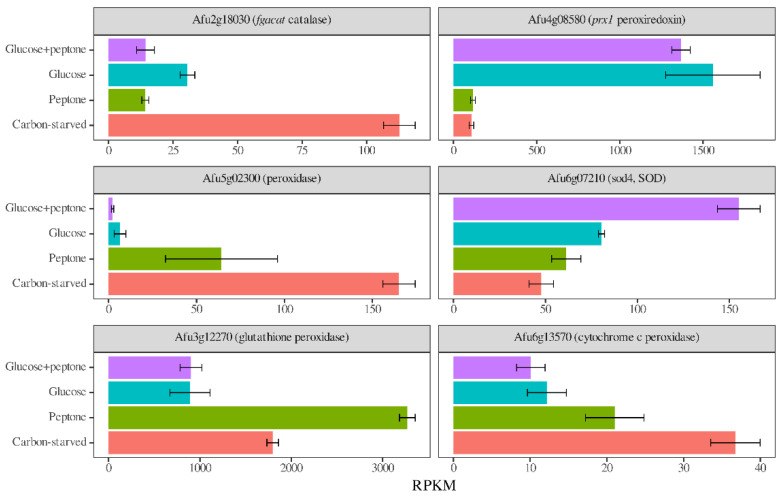
Effects of peptone and glucose withdrawal on the transcriptional activity of selected genes encoding antioxidative enzymes in *Aspergillus fumigatus* Af293. RPKM values generated from the RNAseq experiments (mean ± SD; *n* = 3) are presented.

Despite the redox imbalance caused by carbon stress and the upregulation of certain oxidative stress-related transcription factor genes (e.g., *skn7*, *atfA*, *atfB*, and *atfC*) [39,40] under carbon stress, we did not find bulk upregulation among the genes encoding antioxidative enzymes (Table 3 and Table S4). The only glucose-withdrawal-related enrichments were an upregulation of “catalase, peroxidase and SOD” genes in the absence of peptone and a downregulation of “glutathione, glutaredoxin, thioredoxin system” genes (NADPH, i.e., energy-dependent processes) in the presence of peptone (Table 3 and Table S4). Nevertheless, many genes encoding antioxidative enzymes altered their transcriptional activity after glucose withdrawal or peptone replacement resulting in differences in the profiles of transcriptionally active antioxidative enzyme genes (Figure 6, Table S4). The oxidative stress tolerance was highly dependent on the presence of glucose and peptone on agar plates (Figure S3). In the presence of 0.04 mM MSB, cultures were unable to grow on glucose, showed intensive growth on glucose + peptone, and a moderate growth on peptone (Figure S3).

**Figure 7:**
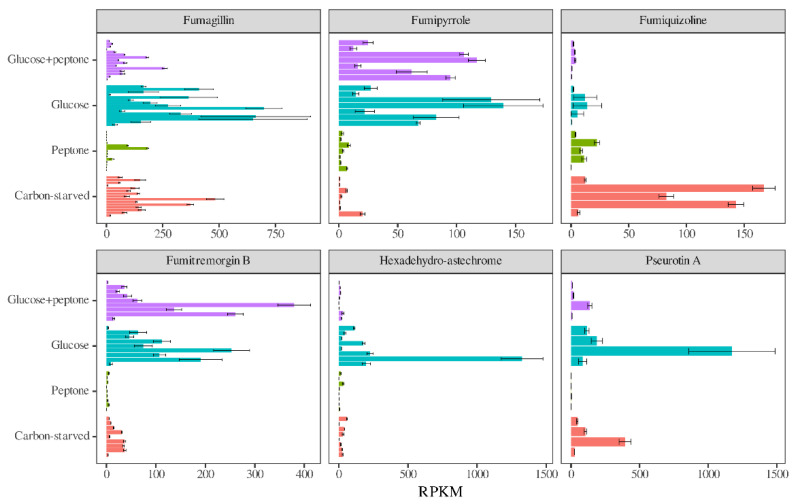
Effects of peptone and glucose withdrawal on the transcriptional activity of genes of selected secondary metabolite clusters in *Aspergillus fumigatus* Af293. RPKM values generated from the RNAseq experiments (mean ± SD; *n* = 3) are presented.

Regarding secondary metabolism, the presence of peptone downregulated several secondary metabolite clusters and cluster genes even in the presence of glucose (Table 4, Table 5 and Table S4). Glucose withdrawal also caused downregulations irrespective of the presence of peptone or caused both upregulations and downregulations in the absence of peptone (Table 4, Table 5 and Table S4). As in the case of antioxidative enzyme genes, the transcriptional behavior of secondary metabolism genes had characteristic features in each of the cultures. As an example, the Fumagillin and Pseurotin A clusters were highly active on glucose and less active under starvation but showed reduced activity in the presence of peptone (Figure 7). The Hexadehydro-astechrome cluster was active only on glucose, while the Fumiquizoline cluster only in carbon-starved cultures (Figure 7). The Fumipyrrole and Fumitremorgin B clusters showed high-transcriptional activity in the presence of glucose irrespective of the presence of peptone (Figure 7).

### 3.6. Carbon Stress Altered the Transcription of Iron Metabolism Genes

Glucose withdrawal downregulated, while the addition of peptone upregulated several genes involved in the siderophore metabolism or the RIA (Table 3 and Table S4). Interestingly, some genes encoding transporters putatively involved in iron transport were upregulated in the absence of glucose (Table 3 and Table S4). Bulk up- or downregulations within the Fe-S cluster assembly genes were not detected, however glucose withdrawal downregulated and the presence of peptone (in the absence of glucose) upregulated genes encoding Fe-S cluster proteins (Table 3 and Table S4).

Heme biosynthesis genes were downregulated by glucose withdrawal (Table 3 and Table S4). Interestingly, peptone downregulated several genes encoding heme proteins, while in the absence of glucose both the upregulated and downregulated heme protein genes were enriched in the appropriate gene sets (Table 3 and Table S4).

In the absence of glucose, there were several known or putative monooxygenase, catalase/peroxidase, steroid and lipid metabolism enzyme genes both among the upregulated and downregulated genes. Genes directly related to growth (e.g., nitrate and sulfate assimilation, respiration genes) occurred typically in the downregulated gene set.

The effect of DFP-induced iron-chelation stress depended on the presence of peptone. In the presence of peptone (irrespective of the presence of glucose) cultures were able to grow on plates containing 3 mM DFP while unable to on glucose as the sole carbon source (Figure S3).

## 4. Discussion

Macroautophagy followed by autolytic cell wall degradation is an efficient way for molds to reutilize their biomass after the colony has exhausted the nutrients in its close environment [10,11,31,41]. Since cell walls represent a substantial portion of fungal biomass, reutilization of this cell component as a carbon energy source is particularly important. The genes involved in cell wall reutilization are upregulated not only under carbon starvation as a “last chance” to obtain nutrients, but also on weak carbon energy sources such as arabinogalactan and lactose in the case of *A. nidulans* [17,32] or peptone in the case of *A. fumigatus* (Table 3 and Table S4). Moreover, N-acetylglucosamine utilization genes are duplicated (completely or partly) not only in Af293 but in approximately 12.4% of the studied *A. fumigatus* genomes, too (Table S6). Reutilization of cell wall polysaccharides can be more important on peptone than on carbohydrates because the low C/N ratio of peptone can limit growth. This may explain why N-acetylglucosamine utilization genes were upregulated in cultures growing on peptone relative to carbon-starved cultures (Table 3 and Tables S2 and S4). The importance of macroautophagy in recycling nitrogen compounds (and metal ions) has been demonstrated by Richie et al. [10]. However, deletion of the *atg1* gene involved in the process of autophagy did not decrease the virulence of *A. fumigatus* [10]. The significance of autolytic cell wall degradation during pathogenesis has not been tested experimentally. Since the availability of carbohydrates is low in the human body and macroautophagy recycles mainly organic nitrogen compounds and lipids, recycling cell wall polysaccharides by autolytic cell wall degradation can be potentially important.

Adaptation to carbon stress includes partial degradation of intracellular GSH pools (i.e., using GSH as a carbon energy source) [31]. The genome of *A. nidulans* contains two γGT genes: the *ggtA* (AN10444) encodes a *Pezizomycotina*-only clade (GGT1 subclade) γGT responsible for both the extracellular and intracellular γGT activities (detected with γ-glutamyl-*p*-nitroanilide as γ-glutamyl donor) of this fungus [17,42] and the AN5658 encodes a putative γGT which belongs to the *Pezizomycotina*–*Saccharomycotina* (GGT3) clade [43]. Deletion of this latter gene did not cause changes in the γGT activities (detected with γ-glutamyl-*p*-nitroanilide substrate) [17]. Although GgtA and the AN5658 protein are upregulated under carbon stress, they are not essential for the degradation of GSH during these conditions. GgtA production (but not AN5658 production) is coregulated with extracellular peptidase formation [17]. It is suggested that GgtA may be involved in the utilization of amino acids and peptides by forming different γ-glutamyl compounds extracellularly to enhance their uptake [17,42]. In contrast to γGTs, the DUG pathway, consisting of two glutamine amidotransferases (DugB and DugC) and a dipeptidase (DugA), is essential to maintain the appropriate intracellular GSH levels in *A. nidulans* [31]. The *dugB* and *dugC* genes are upregulated by carbon stress, however double deletion of the *dugB* and *dugC* genes reduced, but did not prevent, carbon-starvation-induced GSH degradation [31]. The genome of *A. fumigatus* also contains a *Pezizomycotina*-only clade and a *Pezizomycotina*–*Saccharomycotina* clade putative γGT gene (Afu7g04760 and Afu4g13580, respectively). Interestingly, only Afu4g13580 was upregulated by carbon stress and peptone enhanced this upregulation only on glucose (Tables S2 and S4). Upregulation of Afu4g13580 was accompanied with increased intracellular γGT activities but no extracellular γGT activity (and no peptidase secretion) was detected (Figure 3c). Upregulation of any DUG pathway genes was not observed under carbon stress (Table S4), however, the GSH concentration decreased (Figure 2a). One can speculate that the function of *Pezizomycotina*-only clade γGTs (GgtA and Afu7g04760) belongs to the formation and utilization of γ-glutamyl compounds other than GSH, while the *Pezizomycotina*–*Saccharomycotina* clade γGTs (AN5658 and Afu4g13580) are involved in the degradation of GSH. Based on this assumption, the main difference between *A. nidulans* and *A. fumigatus* is that *A. fumigatus* preferred the γGT pathway to the DUG pathway for GSH degradation and did not use its *Pezizomycotina*-only clade γGT to adapt to carbon stress.

The increase in redox imbalance and the decrease in GSH pools (Figure 2) suggest alterations in redox homeostasis elicited by carbon stress in *A. fumigatus*, as was found in *A. nidulans* and *P. chrysogenum* previously [19,31]. These changes were accompanied by altered transcriptions of antioxidative enzyme genes (Table 3 and Table S4 and Figure 6) and altered oxidative stress tolerance in surface cultures (Figure S3). The pattern of transcriptional changes of antioxidative enzyme genes did not show bulk upregulation, only alterations in their transcription: different genes showed high-transcriptional activity on different carbon sources (Table S4 and Figure 6). Concurrent with the decreased nutrient availability, we observed a tendency to upregulate genes encoding catalases, peroxidases (and SODs) and to downregulate genes related to NADPH (energy)-dependent processes (Table S4). In contrast to carbon stress, decreased iron availability tended to downregulate catalase and peroxidase genes (encoding iron containing enzymes) but upregulated SODs and glutathione/glutaredoxin/thioredoxin system genes [27]. Since—due to the immune system—microbes frequently have to cope with oxidative stress, and low levels of both iron and carbon availability in the human body [6,44], setting up the appropriate antioxidative enzyme profile can be challenging during infection. Accordingly, oxidative stress tolerance is usually assumed to be important determinant of virulence. Nevertheless, *A. fumigatus* mutants impaired in the production of one (and sometimes more) antioxidative enzymes or the regulator of their production, did not in many cases show reduced in vivo virulence, despite their decreased in vitro tolerance to oxidative stress [44]. Moreover, *A. fumigatus* oxidative stress tolerance was not superior to other *Aspergillus* strains with less medical importance [45,46]. The rather redundant oxidative stress defense system of *A. fumigatus* partly explains this discrepancy [44]. Our results highlight the extensive effects of nutrition on the transcription of antioxidative enzyme genes (Table 3 and Table S4 and Figure 6) and on stress tolerance (Figure S3). Thus, different antioxidative enzymes are transcribed upon oxidative stress on glucose (often used in vitro) and under glucose limitation (as in vivo), which provides an alternative explanation of why in vitro stress tolerance data sometimes fail to predict the behavior of *A. fumigatus* in vivo.

Transcriptional changes in 34 secondary metabolite gene clusters under carbon stress (Table 4, Table 5 and Table S4 and Figure 7) varied from cluster to cluster. As in earlier studies on carbon and iron limitation or oxidative stress in *A. nidulans* and *A. fumigatus* cultures [27,32,47], our results showed cluster-specific transcriptional changes. Some clusters were upregulated under stress, others were downregulated, and some did not change substantially (Table 4, Table 5 and Table S4 and Figure 7). Here, in general, clusters showed downregulation by the addition of casein peptone or withdrawal of glucose in the presence of peptone (Table 4 and Table S4). However, in the absence of peptone, glucose withdrawal elicited upregulation of some clusters and downregulation of other clusters (Table 4 and Table S4). Some secondary metabolite clusters have been shown to contribute to virulence in *A. fumigatus* [48,49,50]. Among them, the transcriptional activity of the Gliotoxin cluster was low in all our experiments (Table S4). GSH and gliotoxin metabolisms are tightly connected to each other: Gliotoxin increases the oxidative stress tolerance of *A. fumigatus* in an unknown manner [51,52], suggesting a kind of functional link between GSH and this mycotoxin. High-intracellular GSH levels can increase gliotoxin selftoxicity by reducing the gliotoxin disulfide bridge to reactive dithiol [53]. More importantly, biosynthesis of gliotoxin involves a bis-glutathionylation step (conjugation of two GSH molecules to a gliotoxin intermediate) catalyzed by the GliG glutathione S-transferase [54,55]. Therefore, reduced GSH levels are not beneficial for efficient gliotoxin formation. This may explain why gliotoxin cluster genes did not show upregulation under carbon stress (Table S4) where the intracellular GSH concentration was low (Figure 2a). The Fumagillin cluster showed substantial transcriptional activity in both glucose and carbon-starved conditions, while the Hexadehydro-astechrome cluster showed such activity only on glucose (Figure 7 and Table S4). These data suggest that the low availability of glucose in the human body does not in itself promote virulence via secondary metabolism. However, the experimental data that underline the importance of gliotoxin, fumagillin, or hexadehydro-astechrome biosynthesis in pathogenesis [48,49,50] demonstrate that some culturing parameters and stresses other than low glucose levels (e.g., the immune-system-elicited oxidative stress) can activate the production of these mycotoxins in the human body.

Iron withdrawal from microbes as part of “nutritional immunity” is an important strategy of mammals to control infections [56,57]. Therefore, the iron acquisition strategy and iron demand of microbes, including *A. fumigatus* highly influence their success in the human body [57]. Most changes detected in our experiments can be explained by the absence of glucose- or peptone-decreasing growth, and consequently reducing the iron demand (Table 3 and Table S4). Only the transcriptional behavior of heme protein genes was unexpected. Glucose withdrawal enriched these genes both in the upregulated and the downregulated gene sets (Table 3 and Table S4). This behavior calls attention to growth limitation not only reducing iron demand, but also altering how iron is utilized. Previously we found that iron starvation combined with oxidative stress upregulated iron-acquisition processes and also led to alterations in iron utilization [27]. Many genes encoding iron-containing protein were downregulated, but several others were upregulated, which was accompanied with the upregulation of macroautophagy and ubiquitin-dependent protein degradation [27]. Richie et al. [10] also found that macroautophagy can partially cover the iron demand of growth under iron starvation in surface cultures of *A. fumigatus*. Thus, efficient iron uptake processes [57], redistribution of iron within the mycelial network [10,27], and lowered iron demand due to reduced growth can help *A. fumigatus* to efficiently cope with iron limitation stress in the human body. This complex mechanism may lead to redundancy in iron acquisition, which could explain why certain elements of iron metabolism (such as RIA, or macroautophagy) [10,58] are not essential for in vivo virulence, however they have proven to be important in vitro.

## Figures and Tables

**Table 1:** GO terms enriched significantly in the upregulated or downregulated gene sets in glucose-depleted cultures.

	Significantly Enriched GO Terms *	
	Upregulated	Downregulated
Carbon-starved vs. Glucose	Alpha-amino acid catabolic process; Aromatic amino acid family catabolic process; Polysaccharide catabolic process; Cellulose binding; Galacturonan metabolic process; Hemicellulose metabolic process; Pectin catabolic process; Xylan catabolic process; Carbohydrate transport; Hexose transmembrane transport; Glucose transmembrane transport; Secondary metabolic process; Phenol-containing compound biosynthetic process; DNA-binding transcription factor activity; Heme binding.	Cytosolic ribosome; Mitochondrial ribosome; Translation; Ribonucleotide biosynthetic process; Alpha-amino acid biosynthetic process; Alpha-amino acid catabolic process; Glycolysis/Gluconeogenesis; Pentose-phosphate shunt; Acetyl-CoA biosynthetic process; Citrate cycle (TCA cycle); Aerobic respiration; Sulfate assimilation; Sulfur amino acid biosynthetic process; Nitrate transport; Nitrite transport; Vitamin biosynthetic process; Ergosterol biosynthetic process; Fatty acid biosynthetic process; Fungal-type cell wall polysaccharide metabolic process; Glucan biosynthetic process; Iron-sulfur cluster binding; Heme binding; Heme biosynthetic process; Cellular response to iron ion starvation; Siderophore biosynthetic process; Cellular potassium ion homeostasis; Sodium ion transport; Sodium inorganic phosphate symporter activity; Biosynthesis of secondary metabolites; Galactose metabolism; and Serine-type peptidase activity.
Peptone vs. Glucose + peptone	Polysaccharide catabolic process; Carbohydrate transmembrane transporter activity DNA-binding transcription factor activity; Aromatic compound biosynthetic process; and Iron ion binding.	Cytosolic ribosome; Mitochondrial ribosome; Translation; Ribonucleotide biosynthetic process; Alpha-amino acid biosynthetic process; Alpha-amino acid catabolic process; Glycolysis/Gluconeogenesis; Pentose-phosphate shunt; Acetyl-CoA biosynthetic process; Citrate cycle (TCA cycle); Aerobic respiration; Sulfur amino acid biosynthetic process; Vitamin metabolic process; Ergosterol biosynthetic process; Fatty acid biosynthetic process; Heme binding; Heme biosynthetic process; Cellular response to iron ion starvation; Siderophore biosynthetic process; Cellular potassium ion homeostasis; Sodium ion transport; Biosynthesis of secondary metabolites; and Peroxiredoxin activity.
Peptone vs. Glucose	Cellular amino acid catabolic process; Aromatic amino acid family catabolic process; and Ribosome biogenesis.	Cytosolic ribosome; Translation; Ribonucleotide biosynthetic process; Alpha-amino acid biosynthetic process; Alpha-amino acid catabolic process; Glycolysis/Gluconeogenesis; Pentose-phosphate shunt; Acetyl-CoA biosynthetic process; Citrate cycle (TCA cycle); Aerobic respiration; Sulfur compound biosynthetic process; Vitamin metabolic process; Ergosterol biosynthetic process; Lipid biosynthetic process; Fungal-type cell wall organization or biogenesis; Glucan biosynthetic process; Heme binding; Cellular response to iron ion starvation; Cellular potassium ion homeostasis; Sodium ion transport; Inorganic phosphate transmembrane transporter activity; Biosynthesis of secondary metabolites; Galactose metabolism; Glycogen biosynthetic process; Serine-type peptidase activity; Peptide transport; and Peroxiredoxin activity.

* The full list of significantly enriched GO terms is available in Table S3.

**Table 2:** GO terms enriched significantly in the upregulated or downregulated gene sets in peptone-containing cultures.

	Significantly Enriched GO Terms *	
	Upregulated	Downregulated
Peptone vs. Carbon-starved	Ribosome biogenesis; Response to unfolded protein; N-acetylglucosamine catabolic process; and Iron ion homeostasis.	Polysaccharide catabolic process; Cell wall organization or biogenesis; Secondary metabolite biosynthetic process; Melanin metabolic process; and Heme binding.
Glucose + peptone vs. Glucose	Ribosome biogenesis; Translation; Gene expression; Biosynthesis of amino acids; Alpha-amino acid catabolic process; Cysteine and methionine metabolism; Ergosterol biosynthetic process; Fatty acid catabolic process; Cellular response to iron ion starvation; and Siderophore biosynthetic process.	Nitrite transmembrane transporter activity; Nitrate assimilation; Reactive nitrogen species metabolic process; Amino acid transmembrane transporter activity; Glutamate biosynthetic process; Nucleobase transmembrane transporter activity; Urea catabolic process; Maltose metabolic process; Secondary metabolite biosynthetic process; Melanin biosynthetic process; and Heme binding.

* The full list of significantly enriched GO terms is available in Table S3.

**Table 3:** Transcriptional behavior of selected gene groups in carbon-stressed *A. fumigatus* cultures.

Gene Group ^a^	Number of Upregulated/Downregulated Genes				
	Peptone vs. Carbon-Starved	Glucose + Peptone vs. Glucose	Carbon-Starved vs. Glucose	Peptone vs. Glucose + Peptone	Peptone vs. Glucose
Cazyme genes (566)	39/135 ^b^	13/37 ^b^	157 ^b^/109	134 ^b^/146 ^b^	117 ^b^/156 ^b^
Autophagy-related genes (17)	0/1	1/0	2/0	8/1	5/0
Chitinase genes (16)	1/6 ^b^	0/0	5/1	4/3	4/3
Chitine utilization genes (6)	6 ^b^/0	0/0	6 ^b^/0	6 ^b^/0	6 ^b^/0
Glucanase genes (49)	2/16 ^b^	2/3	15 ^b^/14 ^b^	11/17 ^b^	11/19 ^b^
Secreted peptidase genes (36)	2/11 ^b^	1/2	8/14 ^b^	8/17 ^b^	4/19 ^b^
Antioxidative enzyme genes (34)	5/5	2/1	9/8	7/12 ^b^	7/8
Catalases, peroxidases, SODs (15)	2/3	1/1	6 ^b^/2	4/4	4/2
Thioredoxin, glutaredoxin, glutathione systems (19)	3/2	1/0	3/6	3/8 ^b^	3/6
Heme binding protein genes (121)	6/38 ^b^	6/18 ^b^	43 ^b^/36 ^b^	36 ^b^/38 ^b^	32 ^b^/45 ^b^
Heme biosynthesis genes (11)	2/0	0/0	0/6 ^b^	1/6 ^b^	1/4
Fe-S cluster protein genes (43)	7 ^b^/4	1/2	5/18 ^b^	8/16 ^b^	6/16 ^b^
Fe-S cluster assembly genes (15)	1/0	0/0	1/3	2/4	3/3
Fe acquisition genes (30)	9 ^b^/4	8^b^/0	4/16 ^b^	5/16 ^b^	4/16 ^b^
Siderophore metabolism genes (14)	6 ^b^/1	7 ^b^/0	0/11 ^b^	0/11 ^b^	0/10 ^b^
RIA genes (3)	0/1	1/0	0/3 ^b^	0/3 ^b^	0/3 ^b^
other iron transporter genes (8)	2/2	0/0	4/0	4/0	4/1

^a^ Number of genes in the group is presented in parentheses after the name of the group. The whole data set is available in Table S4. ^b^ Significantly enriched (Fisher exact test; *p* < 0.05) in the studied gene set.

**Table 4:** Transcriptional behavior of secondary metabolite clusters and cluster genes in carbon-stressed *A. fumigatus* cultures.

Comparison	Number of Upregulated/Downregulated	
	Clusters ^a^	Genes ^b^
Peptone vs. Carbon-starved	2/10	27/99
Glucose + peptone vs. Glucose	3/8	19/51
Carbon-starved vs. Glucose	9/8	81/86
Peptone vs. Glucose + peptone	4/8	57/104
Peptone vs. Glucose	4/10	50/105

^a^ 34 clusters in total were analyzed. A cluster was regarded as upregulated or downregulated if the upregulated or downregulated cluster genes were significantly enriched (Fisher exact test; *p* < 0.05) in the studied gene set. ^b^ 284 genes in total were analyzed. The whole data set is available in Table S4.

**Table 5:** Transcriptional behavior of selected secondary metabolite cluster genes in carbon-stressed *A. fumigatus* cultures.

Cluster ^a^	Number of Upregulated and Downregulated Genes				
	Peptone vs. Carbon-Starved	Glucose + Peptone vs. Glucose	Carbon-Starved vs. Glucose	Peptone vs. Glucose + Peptone	Peptone vs. Glucose
DHN-melanin cluster (10)	1/7 ^b^	0/4 ^b^	5 ^b^/1	3/2	2/5 ^b^
Endocrocin cluster (9)	2/6 ^b^	1/4 ^b^	7 ^b^/0	5 ^b^/0	5 ^b^/0
Fumagillin cluster (15)	0/13 ^b^	0/11 ^b^	0/10 ^b^	0/13 ^b^	0/13 ^b^
Fumigaclavine C (fga) cluster (11)	0/11 ^b^	0/2	6 ^b^/0	0/3	0/4
Fumipyrrole cluster (7)	2/1	0/0	0/7 ^b^	0/7 ^b^	0/7 ^b^
Fumiquizoline cluster (5)	0/5 ^b^	0/3 ^b^	5 ^b^/0	2/1	0/1
Fumitremorgin B (ftm) cluster (9)	0/8 ^b^	0/1	0/8 ^b^	0/8 ^b^	0/8 ^b^
Gliotoxin (gli) cluster (12)	3 ^b^/0	0/7 ^b^	0/10 ^b^	2/6 ^b^	1/9 ^b^
Hexadehydro-astechrome cluster (8)	0/7 ^b^	0/7 ^b^	0/7 ^b^	2/3	0/7 ^b^
Pseurotin A cluster (4)	0/4 ^b^	0/4 ^b^	0/4 ^b^	0/4 ^b^	0/4 ^b^
Siderophore cluster (18)	6 ^b^/0	9 ^b^/2	2/9 ^b^	3/10 ^b^	1/9 ^b^

^a^ Number of genes in the cluster is presented in parentheses after the name of the cluster. The whole data set is available is Table S4. ^b^ Significantly enriched (Fisher exact test; *p* < 0.05) in the studied gene set.

## Data Availability

Transcriptome data sets are available in the Gene Expression Omnibus database (GEO; http://www.ncbi.nlm.nih.gov/geo/; accessed on 15 November 2022) with the following accession number: GSE216000.

## Supplementary Materials

The following supporting information can be downloaded at: https://www.mdpi.com/article/10.3390/jof8111226/s1, Figure S1: Morphology of *Aspergillus fumigatus* Af293 cultures; Figure S2: Position of N-acetyl glucosamine transporter, glucosamine-6-phosphate deaminase, and N-acetylglucosamine-6-phosphate deacetylase genes in the genome of *A. fumigatus* Af293 and A1163; Figure S3: Effect of menadione sodium bisulfite (MSB) and deferiprone (DFP) on the growth of *A. fumigatus* Af293; Table S1: Primer pairs and *Aspergillus fumigatus* genomes used in the study; Table S2: RT-qPCR data of selected genes; Table S3: Results of the gene set enrichment analyses; Table S4: Transcription data generated by RNA sequencing of selected groups of functionally related genes; Table S5: Transcriptional activity of the Afu8g04070-Afu8g04130 and Afu1g00410-Afu1g00480 gene sets according to the RNA sequencing data; Table S6: Occurrence of Afu1g00410-Afu1g480 and Afu8g04070-Afu8g04130 gene orthologues in the studied *A. fumigatus* genomes.
