# Supplementary material for: Stress Responses Elicited by Glucose Withdrawal in Aspergillus fumigatus
Source: J Fungi (Basel). 2022 Nov 21;8(11):1226. doi: 10.3390/jof8111226 (PMC9692504; doi:10.3390/jof8111226)
Supplement: Supplementary file 1 [file jof-08-01226-s001.zip › Figure. S1.pptx]

## Slide 1
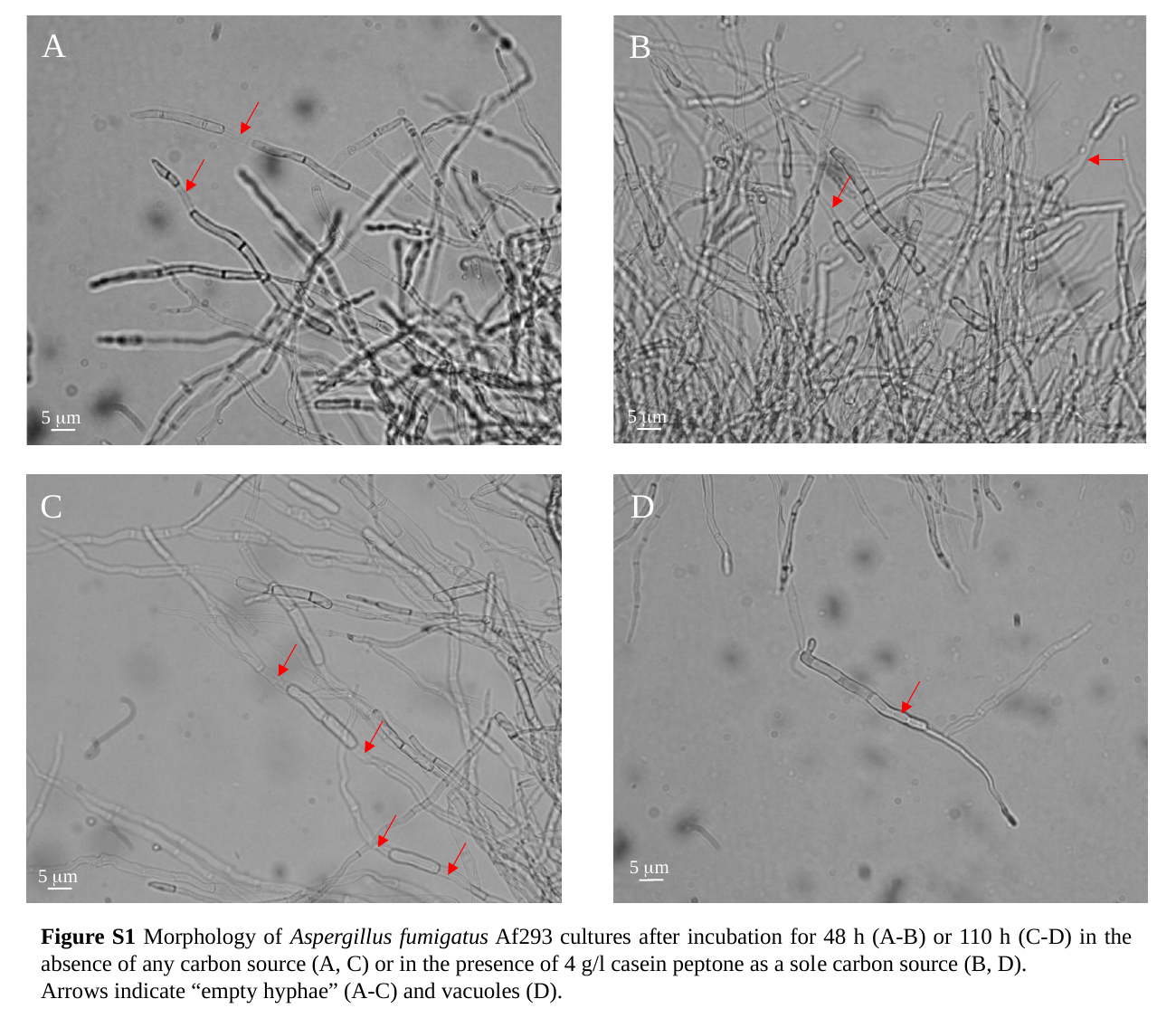

A
B
5 mm
5 mm
C
D
5 mm
5 mm
Figure S1 Morphology of Aspergillus fumigatus Af293 cultures after incubation for 48 h (A-B) or 110 h (C-D) in the absence of any carbon source (A, C) or in the presence of 4 g/l casein peptone as a sole carbon source (B, D).
Arrows indicate “empty hyphae” (A-C) and vacuoles (D).
