# Supplementary material for: Stress Responses Elicited by Glucose Withdrawal in Aspergillus fumigatus
Source: J Fungi (Basel). 2022 Nov 21;8(11):1226. doi: 10.3390/jof8111226 (PMC9692504; doi:10.3390/jof8111226)
Supplement: Supplementary file 1 [file jof-08-01226-s001.zip › Figure. S2.pptx]

## Slide 1
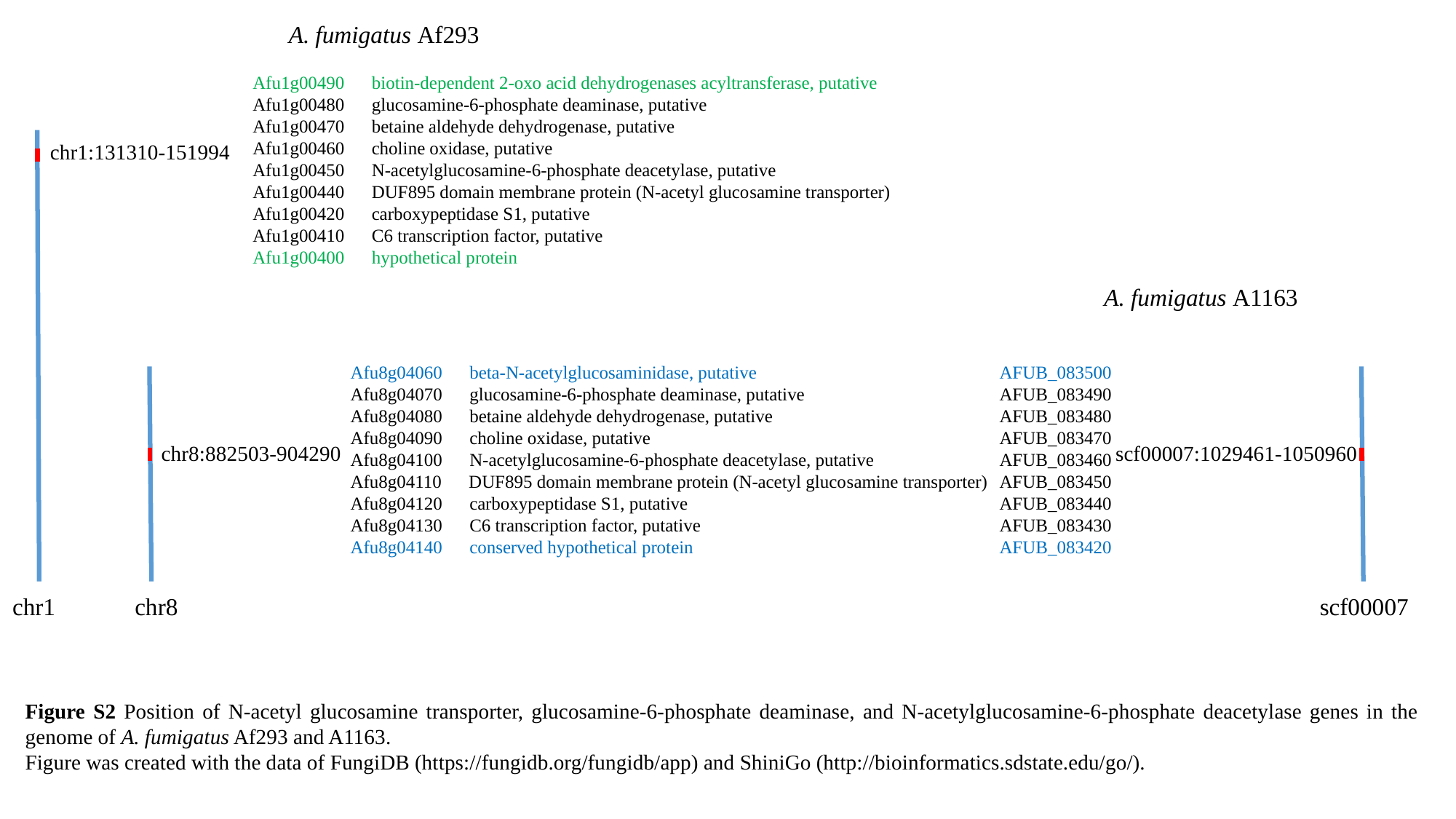

A. fumigatus Af293
Afu1g00490 biotin-dependent 2-oxo acid dehydrogenases acyltransferase, putative
Afu1g00480 glucosamine-6-phosphate deaminase, putative
Afu1g00470 betaine aldehyde dehydrogenase, putative
Afu1g00460 choline oxidase, putative
Afu1g00450 N-acetylglucosamine-6-phosphate deacetylase, putative
Afu1g00440 DUF895 domain membrane protein (N-acetyl glucosamine transporter)
Afu1g00420 carboxypeptidase S1, putative
Afu1g00410 C6 transcription factor, putative
Afu1g00400 hypothetical protein
chr1:131310-151994
A. fumigatus A1163
Afu8g04060 beta-N-acetylglucosaminidase, putative
Afu8g04070 glucosamine-6-phosphate deaminase, putative
Afu8g04080 betaine aldehyde dehydrogenase, putative
Afu8g04090 choline oxidase, putative
Afu8g04100 N-acetylglucosamine-6-phosphate deacetylase, putative
Afu8g04110 DUF895 domain membrane protein (N-acetyl glucosamine transporter)
Afu8g04120 carboxypeptidase S1, putative
Afu8g04130 C6 transcription factor, putative
Afu8g04140 conserved hypothetical protein
AFUB_083500
AFUB_083490
AFUB_083480
AFUB_083470
AFUB_083460
AFUB_083450
AFUB_083440
AFUB_083430
AFUB_083420
chr8:882503-904290
scf00007:1029461-1050960
chr1
chr8
scf00007
Figure S2 Position of N-acetyl glucosamine transporter, glucosamine-6-phosphate deaminase, and N-acetylglucosamine-6-phosphate deacetylase genes in the genome of A. fumigatus Af293 and A1163.
Figure was created with the data of FungiDB (https://fungidb.org/fungidb/app) and ShiniGo (http://bioinformatics.sdstate.edu/go/).
