# Supplementary material for: Stress Responses Elicited by Glucose Withdrawal in Aspergillus fumigatus
Source: J Fungi (Basel). 2022 Nov 21;8(11):1226. doi: 10.3390/jof8111226 (PMC9692504; doi:10.3390/jof8111226)
Supplement: Supplementary file 1 [file jof-08-01226-s001.zip › Figure. S3.pptx]

## Slide 1
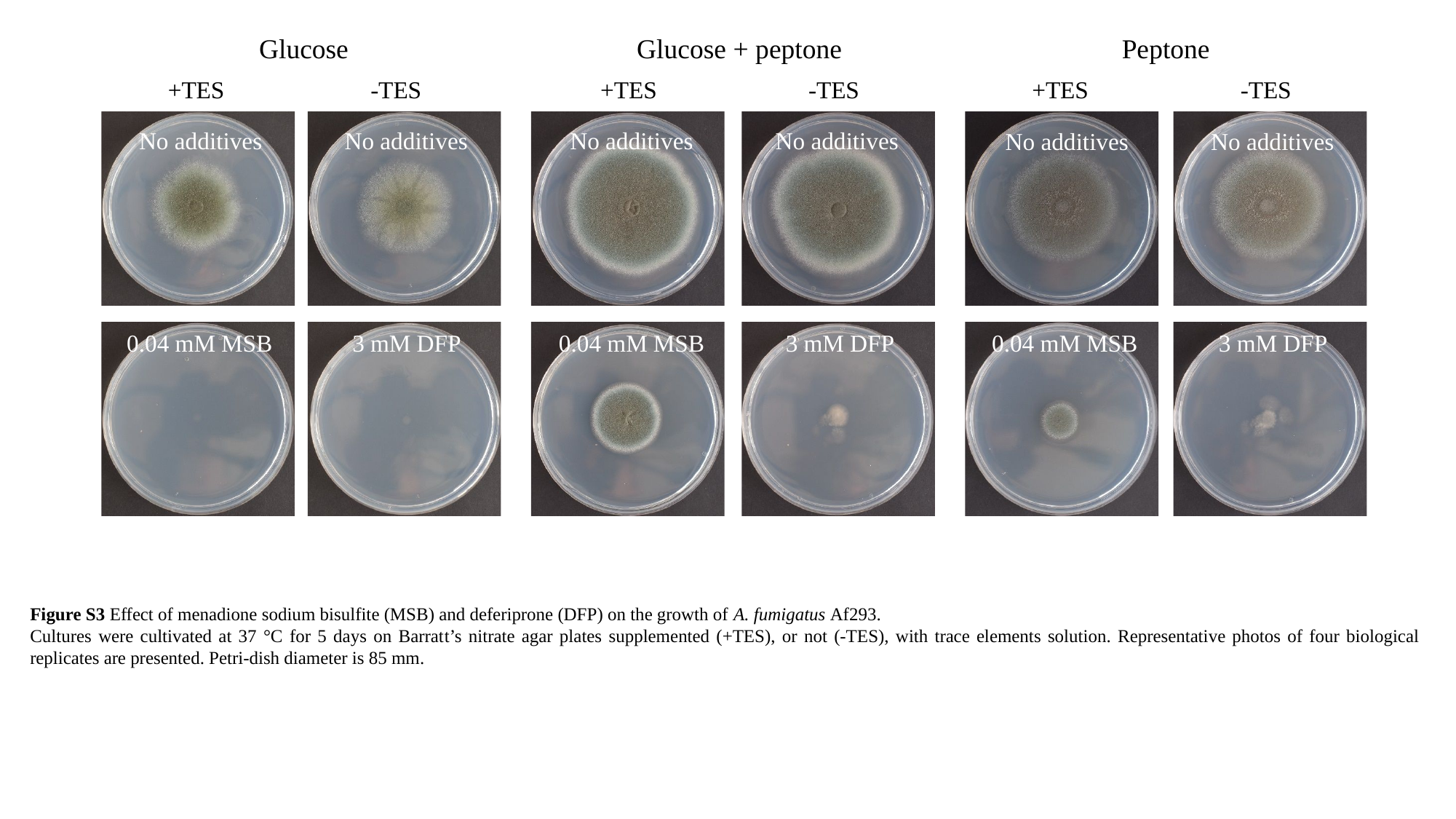

Glucose
Glucose + peptone
Peptone
+TES
-TES
+TES
-TES
+TES
-TES
No additives
No additives
No additives
No additives
No additives
No additives
0.04 mM MSB
3 mM DFP
0.04 mM MSB
3 mM DFP
0.04 mM MSB
3 mM DFP
Figure S3 Effect of menadione sodium bisulfite (MSB) and deferiprone (DFP) on the growth of A. fumigatus Af293.
Cultures were cultivated at 37 °C for 5 days on Barratt’s nitrate agar plates supplemented (+TES), or not (-TES), with trace elements solution. Representative photos of four biological replicates are presented. Petri-dish diameter is 85 mm.
