# Supplementary material for: Stress Responses Elicited by Glucose Withdrawal in Aspergillus fumigatus
Source: J Fungi (Basel). 2022 Nov 21;8(11):1226. doi: 10.3390/jof8111226 (PMC9692504; doi:10.3390/jof8111226)
Supplement: Supplementary file 1 [file jof-08-01226-s001.zip › Table S1.pdf]

Table S1 Primer pairs and *Aspergillus fumigatus* genomes used in the study.

Primer pairs used in RT-qPCR assays

Afu1g04260

Forward:5'-CGTCTTTGTACCAGTGTC-3'

Reverse:5'-CATAGCTCTGTGACGTTC-3'

Afu1g00440

Forward:5'-GATGAAGAACCCTACCTG-3'

Reverse:5'-CTGTTGAGAGACCAGGTAC-3'

Afu1g00450

Forward:5'-GTGACTTCATACTTGCCTAC-3'

Reverse:5'-CTCTTTGCCATAACACTC-3'

Afu1g12940

Forward:5'-GCCTTTGGACTTGTCTGCTC-3'

Reverse:5'-GTCTGTCCCAAGGAGCTCTG-3'

Afu1g14550

Forward:5'-CAGAAGAAAGCGGCGGAAG-3'

Reverse:5'-AGGGTCGTGTTGAAAGCATC-3'

Afu2g07680

Forward:5'-TCTTCTGTGTGTTGGGTTTGG-3'

Reverse:5'-TTCTGGTGGAGGTAGTTGAG-3'

Afu3g11280

Forward:5'-GGGTGGTTACAAGTCTATC-3'

Reverse:5'-CAACTCTCAGAAACACCTC-3'

Afu4g13580

Forward:5'-GCGATACAGTCTACTTCG-3'

Reverse:5'-AGAGTGAAGAGAGCCATC-3'

Afu5g03800

Forward:5'-TTGACTGGCGGCACATTC-3'

Reverse:5'-TGGAGGGAGGCTTGGTTTC-3'

Afu5g03920

Forward:5'-ATTCCACCACGACCAAAACC-3'

Reverse:5'-CACCGATACCTCCTTCTCCAAC-3'

Afu5g11260

Forward:5’-GAGACCACATCATCCACCG-3’

Reverse:5’-TTCAAGTTCAGACACGCCAG-3’

A. fumigatus genomes listed by their NCBI (https://www.ncbi.nlm.nih.gov/home/genomes/) *accession number analyzed in the study*

DRR015087
DRR015088
DRR015089
DRR015092
DRR015093
DRR015094
DRR015095
DRR015096
DRR015097
DRR015098
DRR015099
DRR015100
DRR015101
DRR015102
DRR015105
DRR015106
DRR015107
DRR015108
DRR015109
DRR015110
DRR015111
DRR015112
DRR017535
DRR017536
DRR017537
DRR017538
DRR017539
DRR017540
DRR017541

DRR017542
DRR017543
DRR017544
DRR017545
DRR017546
DRR017547
DRR017548
DRR017549
DRR017550
DRR017551
DRR017552
DRR017553
DRR017554
DRR017555
DRR017556
DRR017557
DRR017558
DRR017559
DRR017560
DRR017561
DRR022915
DRR022916
DRR022917
DRR022918
DRR022919
DRR022920
DRR022921
DRR022922
DRR022923
DRR022924
DRR022925
DRR022926
DRR022927
DRR146814

DRR290036
DRR295873
DRR295874
DRR295875
ERR2863830
ERR2864483
ERR2864484
ERR2864485
ERR769499
ERR769500
ERR769501
ERR769502
ERR769503
ERR769504
ERR769505
ERR769506
ERR769507
ERR769508
ERR769509
ERR769510
ERR769511
ERR769512
ERR769513
ERR769514
ERR769515
ERR769516
ERR769517
ERR769518
ERR769519
ERR769520
ERR769521
SRR10592629
SRR10592630
SRR10592631

SRR10592632
SRR10592633
SRR10997277
SRR10997288
SRR10997299
SRR10997300
SRR12894193
SRR12894194
SRR12894195
SRR12894196
SRR12894197
SRR12894198
SRR12894199
SRR12894200
SRR12894201
SRR12894202
SRR12894203
SRR12894204
SRR2954803
SRR4002443
SRR4002444
SRR5676586
SRR5676587
SRR5676588
SRR5676589
SRR5676590
SRR5676591
SRR5676592
SRR5676593
SRR617720
SRR617721
SRR617722
SRR617723
SRR617724

SRR617725
SRR617726
SRR617727
SRR617728
SRR617729
SRR617731
SRR617733
SRR617734
SRR617735
SRR617736
SRR617737
SRR617738
SRR617739
SRR617742
SRR617744
SRR617745
SRR6434917
SRR6434918
SRR7418922
SRR7418923
SRR7418924
SRR7418925
SRR7418926
SRR7418927
SRR7418928
SRR7418929
SRR7418930
SRR7418932
SRR7418933
SRR7418934
SRR7418935
SRR7418936
SRR7418937
SRR7418938

SRR7418939
SRR7418940
SRR7418941
SRR7418942
SRR7418943
SRR7418944
SRR7418945
SRR7418946
SRR7418947
SRR7418948
SRR7418949
SRR8759697
SRR8759698
SRR8759699
SRR8759700
SRR8759701
SRR8759702
SRR8759703
SRR8759704
SRR8759705
SRR8759706
SRR8759707
SRR8759708
SRR8759709
SRR9067511
SRR9265315
SRR9265316
SRR9265317
SRR9265318
