# Supplementary material for: Stress Responses Elicited by Glucose Withdrawal in Aspergillus fumigatus
Source: J Fungi (Basel). 2022 Nov 21;8(11):1226. doi: 10.3390/jof8111226 (PMC9692504; doi:10.3390/jof8111226)
Supplement: Supplementary file 1 [file jof-08-01226-s001.zip › Table S5.pdf]

Gene ID	Number of reads[a]			
	Carbon-starved (23.2 ± 2.4 million)[b]	Peptone (21.4 ± 3.5 million)	Glucose (20.3 ± 1.6 million)	Glucose+peptone (19.4 ± 3.0 million)
Afu1g00480 (11/1092)[c]	86 ± 9	305 ± 136	1 ± 1	5 ± 1
Afu8g04070 (10/1077)	59 ± 7	229 ± 123	2 ± 1	2 ± 1
both	1023 ± 92	3390 ± 1657	27 ± 8	38 ± 9
Afu1g00470 (17/1500)	304 ± 22	350 ± 49	156 ± 62	208 ± 45
Afu8g04080 (17/1500)	766 ± 68	1006 ± 198	374 ± 76	601 ± 84
both	979 ± 131	1207 ± 197	418 ± 70	742 ± 91
Afu1g00460 (21/1524)	2681 ± 193	5007 ± 1077	1186 ± 133	3842 ± 581
Afu8g04090 (126/1629)	1169 ± 119	2127 ± 430	503 ± 76	1538 ± 274
both	9822 ± 236	24172 ± 10238	2060 ± 446	1933 ± 291
Afu1g00450 (10/1293)	117 ± 5	656 ± 422	5 ± 5	4 ± 2
Afu8g04100 (10/1293)	768 ± 54	3467 ± 1851	18 ± 10	12 ± 11
both	1371 ± 156	6397 ± 3477	38 ± 20	28 ± 11
Afu1g00440 (26/1533)	627 ± 49	1609 ± 687	80 ± 12	90 ± 12
Afu8g04110 (26/1533)	3819 ± 127	10189 ± 5280	168 ± 29	114 ± 15
both	1585 ± 29	4124 ± 2050	82 ± 15	81 ± 13
Afu1g00420 (21/1614)	15 ± 4	20 ± 9	32 ± 9	23 ± 5
Afu8g04120 (63/1656)	12745 ± 373	29537 ± 10778	3290 ± 379	5963 ± 858
both	15 ± 3	16 ± 12	24 ± 2	24 ± 5
Afu1g00410 (137/2550)	709 ± 30	1296 ± 306	278 ± 50	151 ± 43
Afu8g04130 (245/2658)	9915 ± 142	24580 ± 9582	2336 ± 218	5427 ± 789
both	544 ± 6	927 ± 212	237 ± 39	125 ± 29

Table S5 Transcriptional activity of the Afu8g04070-Afu8g04130 and Afu1g00410-Afu1g00480 gene sets according to the RNA sequencing data.

[a] - Mean ± SD of reads that can be aligned only to one of the two genes or to both genes calculated from the three parallel samples.

[b] – Mean ± SD of reads aligned to the transcript sequences in the three parallel samples.

[c] – Number of unique nucleotides in the transcript/length of the transcript. Transcript sequences were downloaded from FungiDB (https://fungidb.org/fungidb/app).
